# Evaluation of the anatomical variations of the coronary venous system in patients with coronary artery calcification using 256-slice computed tomography

**DOI:** 10.1371/journal.pone.0242216

**Published:** 2020-11-18

**Authors:** Wei Bai, Xiao Xu, Haixia Ji, Jing Liu, Heng Ma, Haizhu Xie, Jianjun Dong, Chunjuan Sun, Yinghong Shi, Kaili Che, Meijie Liu, Yingkun Guo

**Affiliations:** 1 Yantai Yuhuangding Hospital, Qingdao University, Yantai, Shandong Province, China; 2 Department of Radiology, Key Laboratory of Obstetric & Gynecologic and Pediatric Diseases and Birth Defects of Ministry of Education, West China Second University Hospital, Sichuan University, Chengdu, Sichuan Province, China; 3 Binzhou Medical University, Yantai, Shandong Province, China; University Medical Center Groningen, University of Groningen, NETHERLANDS

## Abstract

The factors that determine the anatomical variations of the coronary venous system (CVS) are poorly understood. The objective of this study was to evaluate the anatomical variations of the CVS in patients with coronary artery calcification. 196 patients underwent non-contrast CT and coronary CT angiography using 256-slice CT. All subjects were divided into four groups based on their coronary artery calcium score (CACS): 50 patients with CACS = 0 Agatston unit (AU), 52 patients with CACS = 1–100 AU, 44 patients with CACS = 101–400 AU, and 50 patients with CACS > 400 AU. The presence of the following cardiac veins was evaluated: the coronary sinus (CS), great cardiac vein (GCV), posterior interventricular vein (PIV), posterior vein of the left ventricle (PVLV), left marginal vein (LMV), anterior interventricular vein (AIV), and small cardiac vein (SCV). Vessel diameters were also measured. We found that the CS, GCV, PIV, and AIV were visualized in all patients, whereas the PVLV and LMV were identified in a certain proportion of patients: 98% and 96% in the CACS = 0 AU group, 100% and 78.8% in the CACS = 1–100 AU group, 93.2% and 77.3% in the CACS = 101–400 AU group, and 98% and 78% in the CACS > 400 AU group, respectively. The LMV was less often identified in the last three groups than in the first group (p < 0.05). The frequency of having either one PVLV or LMV was higher in the last three groups than in the first group (p < 0.05). No significant differences in vessel diameters were observed between the groups. It was concluded that patients with coronary artery calcification were less likely to have the LMV, which might hamper the left ventricular lead implantation in cardiac resynchronization therapy.

## Introduction

Over the past decade, our knowledge of the coronary venous system (CVS) has increased because of advances in interventional cardiac procedures, such as cardiac resynchronization therapy (CRT), percutaneous mitral annuloplasty, and radiofrequency catheter ablation [1–4]. The anatomical variations of the CVS have become one of the most investigated issues, because a knowledge of the CVS anatomy is necessary and significative before interventional processes within CVS, which can increase the success rate of the operations [5]. However, the factors that determine the anatomical variations of the CVS are poorly understood.

The anatomy of the CVS in patients with myocardial infarction (MI) or heart failure varies [6, 7]. Van de Veire et al. [7] studied 100 subjects via 64-slice computed tomography (CT) and found that the frequency of having no left marginal veins (LMV) was higher in patients with previous MI than in patients without MI. MI occurs when the blood flow to the heart is decreased or completely blocked. The complete blockage of the coronary artery is often caused by the rupture of an atherosclerotic plaque which is usually the underlying mechanism of a MI. Coronary artery calcification is an important coronary atherosclerosis sign, which is closely related to the severity of coronary atherosclerosis [8]. In an interesting study, Mlynarska et al. [9] used a 64-slice CT scan to assessed the relationship between variation in the coronary veins and the extent of coronary artery calcium score (CACS) that can reflect the severity of coronary atherosclerosis. They found the subjects with higher CACS (> 100 AU) had more visible coronary veins (≥5) than the groups with lower and no CACS. However, they didn't analyze the specific variation in each vein. To our best knowledge, studies about the effect of coronary artery calcification on the anatomy of the CVS by using 256-slice CT have not been conducted. Therefore, our study aimed to assess the anatomical variations of the CVS in patients with coronary artery calcification in detail by using 256-slice CT.

## Materials and methods

### Study population

The anatomy of the CVS and CACS were retrospectively studied in 196 consecutive patients (96 females and 100 males; mean age: 63.3 ± 9.9 years). All patients underwent non-contrast CT and coronary CT angiography (CTA) between June 2018 and September 2019 for suspected coronary artery disease (CAD). These patients were divided into four groups based on the CACS: 50 (25.5%) patients without calcification (CACS = 0 AU, Agatston unit), 52 (26.5%) patients with minimal to mild calcification (CACS = 1–100 AU), 44 (22.4%) patients with moderate calcification (CACS = 101–400 AU), and 50 (25.5%) patients with severe calcification (CACS > 400 AU).

Patients with the following medical conditions were excluded from our study: atrial fibrillation, second- or third-degree atrioventricular block, renal insufficiency, creatinine level above 2 mg/dL, known allergy to iodine-containing contrast agents, and pregnancy. The study protocol was approved by the ethics committee of Yantai Yuhuangding Hospital. And the need for written informed patient consent was waived, because this was a retrospective study of coronary CT angiography performed for clinical purposes.

### Coronary CT angiography protocol

Non-contrast CT and coronary CTA were conducted on the same day by using 256-slice CT (Brilliance iCT, Phillips, Cleveland, Ohio, USA). On the examination day, the patients were evaluated by measuring their heart rate and blood pressure 1 h prior to CT acquisition. An intravenous β-blocker was given to decrease patients’ heart rate if it was higher than 90 bpm.

A low-dose non-contrast CT scan was performed to assess the CACS. Then, the coronary CTA was performed with the following scan parameters: tube voltage, 100 kVp; effective tube current time product, 700 mAs; pitch, 0.18; detector configuration, 128 × 0.625 mm; and rotation time, 270 ms. An average of 75 ml of contrast agent (Ultravist 370, Bayer Schering Pharma AG, Berlin. Germany) was injected in the antecubital vein at a rate of 5 ml/s by using a dual-head injector. First, 60 ml of contrast agent (average) was administrated. Then 30 ml of a 1:1 mixture of contrast and saline was given; Lastly, 30 ml of saline was given. Retrospective electrocardiogram triggering was conducted to scan 45% and 75% of the RR interval. Automatic bolus tracking (Bolus Pro, Philips Healthcare, Cleveland, OH, USA) was performed in the ascending aorta, and coronary CTA scanning was automatically started 6 s after the predetermined threshold level of 180 Hounsfield units (HU) was reached.

### Image reconstruction and analysis

**Fig 1:**
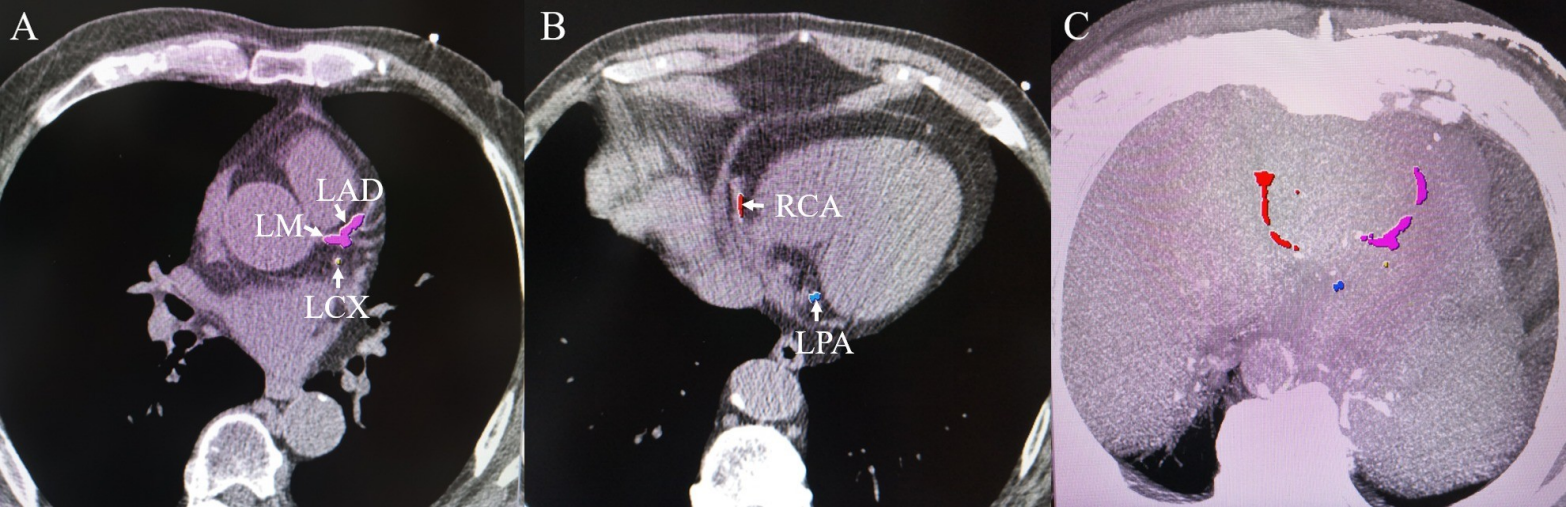
Example of the assessment of the coronary artery calcium score on axial images from the non-contrast CT. (A) and (B) Axial images; (C) superimposed axial images. Extensive calcification observed in the left main coronary artery (LM, pink), left anterior descending artery (LAD, pink), left circumflex coronary artery (LCX, yellow), right coronary artery (RCA, red), and posterior branch of the left ventricle artery (LPA, blue).

**Fig 2:**
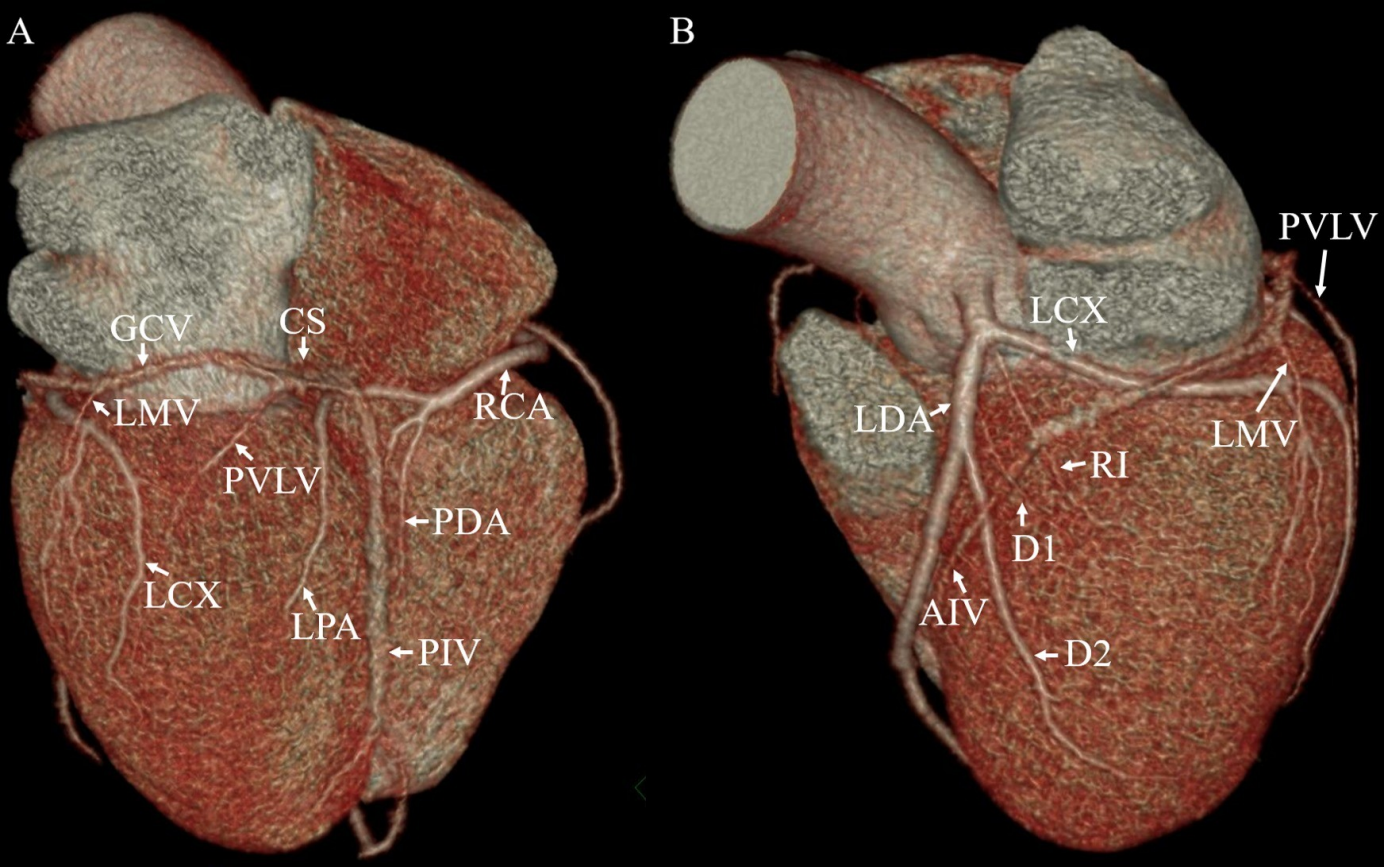
Volume-rendering image provides an overview of the evaluated coronary veins. (A) Posterior view; (B) anterolateral view. The coronary sinus (CS) and great cardiac vein (GCV) run along the atrioventricular groove. The posterior interventricular vein (PIV) is the first tributary of the CS that courses in the posterior interventricular groove. The other tributaries of the CS are the posterior vein of the left ventricle (PVLV) and the left marginal vein (LMV). The great cardiac vein (GCV) then continues as the anterior interventricular vein (AIV) in the anterior interventricular groove. Also note the left anterior descending artery (LAD), left circumflex coronary artery (LCX), ramus intermedius (RI), diagonal branches (D1, D2), posterior descending artery (PDA), posterior branch of left ventricle artery (LPA) and right coronary artery (RCA).

The collected images were transferred to two designated workstations (IntelliSpace Portal, Cleveland, Ohio, USA; GE, Advantage Workstation, version 4.6) and analyzed. The CACS was assessed by using images with 2.5 mm slice thickness from non-contrast CT (Fig 1). In this procedure, pixels exceeding 130 HU were identified and encircled in the course of a coronary artery and calculated with the Agatston method [10]. For the anatomic observation of CVS, the following cardiac veins were evaluated on the collected images from the coronary CTA: the coronary sinus (CS), great cardiac vein (GCV), posterior interventricular vein (PIV), posterior vein of the left ventricle (PVLV), left marginal vein (LMV), anterior interventricular vein (AIV), and small cardiac vein (SCV) (Fig 2). Multiplanar reformatting was conducted to determine the size of the ostium of the CS in the anteroposterior and superoinferior direction. The proximal diameter of the GCV and the starting diameters of the PIV, PVLV, LMV and AIV were measured.

### Statistical analysis

Continuous variables were expressed as mean ± standard deviation. Differences between the groups were examined in terms of continuous variables via ANOVA. Categorical variables were expressed as absolute numbers (percentage) and analyzed with a χ^2^ test. Data with *P* value < 0.05 were considered statistically significant. Statistical analyses were performed by using SPSS version 23.0.

## Results

### Baseline characteristics

The baseline characteristics of the patients included in this study are presented in Table 1. The patients with coronary artery calcification in the last three groups were older than those who had no detectable CACS in the first group and the hemodynamic parameters, including EDV, ESV, and cardiac output of the former were higher than those of the latter. The frequency of cardiac risk factors, including smoking, diabetes, hypertension, and hypercholesterolemia was also higher in the former than in the latter. The CACS > 400 AU group of patients who had higher CACS was mostly composed of men.

**Table 1:** Baseline characteristics of the study population.

CASS	0 AU (n = 50)	1–100 AU (n = 52)	101–400 AU (n = 44)	> 400 AU (n = 50)	*P*
Age, years	55.7 ± 8.7	63.7 ± 9.8	66.8 ± 8.4	67.4 ± 8.3	<0.01
Male, %	50	36.5	47.7	70	0.08
Hemodynamic parameters					
Ejection fraction, %	65.6 ± 9.9	66.9 ± 3.3	65.7 ± 5.4	66.1 ± 5.2	0.756
EDV, ml	122.5 ± 31.7	133.9 ± 37.8	135.5 ± 42.0	149.2 ± 45.9	0.021
ESV, ml	80.3 ± 18.1	90.0 ± 26.2	90.4 ± 26.2	98.2 ± 28.6	0.013
Cardiac output, L/min	5.9 ± 1.7	6.4 ± 2.0	6.0 ± 1.9	7.1 ± 2.2	0.016
Cardiovascular risk factors					
Heart Rate, bpm	77.8 ± 10.6	75.3 ± 10.9	74.2 ± 10.9	76.5 ± 11.0	0.449
Smoking, %	17.9	20.4	25.6	43.8	0.024
Diabetes, %	12.5	14.3	29.5	41.7	0.003
Hypertension, %	40.0	53.1	61.4	68.8	0.046
Hypercholesterolemia, %	32.5	53.2	27.9	31.1	0.049

EDV = left ventricular end-diastolic volume; ESV = left ventricular end-systolic volume.

### Visualization of the coronary veins and CACS

The CS, GCV, PIV and AIV were visualized in all the patients (100%). Table 2 shows the visualization of the PVLV and LMV in the four groups. Statistical differences were observed in cases without LMV visualized. The LMV was less often identified in the last three groups (CACS ≥ 1 AU) than in the CACS = 0 AU group (Pearson Chi-squared = 8.381; p = 0.039). No statistical differences were observed in the visualization of the PVLV. The frequency of having either one PVLV or LMV and either PVLV or LMV (≥1) were higher in the last three groups than in the CACS = 0 AU group (Pearson Chi-squared = 8.430 and 9.208; p = 0.038 and 0.027). The percentages of SCV (P>0.05) observed varied between groups: 7 (14.0%) subjects in the CACS = 0 AU group, 2 (3.8%) subjects in the CACS = 1–100 AU group, 7 (15.9%) subjects in the CACS = 101–400 AU group and 5 (10%) subjects in the CACS > 400 AU group.

**Table 2:** Visualization of the PVLV and LMV.

CASS	0 AU (n = 50)	1–100 AU (n = 52)	101–400 AU (n = 44)	> 400 AU (n = 50)	*p*
PVLV	49 (98%)	52 (100%)	41 (93.2%)	49 (98%)	0.195
1 PVLV	36 (72%)	39 (75%)	26 (59.1%)	34 (68%)	0.373
2 or 3 PVLVs	13 (26%)	13 (25%)	15 (34.1%)	15 (30%)	0.754
LMV	48 (96%)	41 (78.8%)	34 (77.3%)	39 (78%)	0.039
1 LMV	42 (84%)	38 (73.1%)	30 (68.2%)	35 (70%)	0.283
2 or 3 LMVs	6 (12%)	3 (5.8%)	4 (9.1%)	4 (8%)	0.731
Either one PVLV or LMV	1 (2%)	9 (17.3%)	8 (18.2%)	10 (20%)	0.038
Either PVLV or LMV (≥1)	3(6%)	11(21.2%)	13(29.5%)	12(24%)	0.027

### Quantitative measurement and CACS

The diameters of the CS ostium and its tributaries are presented in Table 3. The mean diameter of the CS ostium in the anteroposterior direction in all the patients was smaller than that in the superoinferior direction. No significant differences regarding the diameters of the CS ostium and its tributaries were noted in these groups.

**Table 3:** Diameters of the CS ostium and its tributaries (mm).

CASS	0 AU (n = 50)	1–100 AU (n = 52)	101–400 AU(n = 44)	> 400 AU (n = 50)	*p*
CSO anteroposterior	9.1 ± 2.6	8.9 ± 2.1	9.2 ± 2.6	9.6 ± 2.4	NS
CSO superoinferior	12.6 ± 2.3	12.0 ± 2.1	11.8 ± 3.0	12.6 ± 2.3	NS
GCV proximal	9.1 ± 2.2	9.0 ± 1.9	9.3 ± 2.3	9.4 ± 2.0	NS
PIV	5.3 ± 1.4	5.4 ± 1.2	5.3 ± 1.1	5.6 ± 1.4	NS
PVLV	3.0 ± 1.2	3.2 ± 1.2	3.2 ± 1.1	3.1 ± 1.1	NS
LMV	2.4 ± 0.9	2.3 ± 0.5	2.3 ± 0.8	2.4 ± 0.7	NS
AIV	3.8 ± 0.8	4.0 ± 0.9	3.9 ± 0.8	4.2 ± 1.0	NS

## Discussion

This study was the first to evaluate the relationship between the CVS and coronary artery calcification by using 256-slice CT. The incidence of LMV in patients with coronary artery calcification was lower than in patients without coronary artery calcification. The frequency of having either one PVLV or LMV in the patients with coronary artery calcification was higher than that of the patients without calcification. The decreased prevalence of the CS tributaries might hamper the access of patients who are considered for CRT to optimal left ventricle (LV) lead positioning. Before CRT implantation, the detailed images of the coronary vein anatomy in the target area could be obtained by using 256-slice CT and sequentially provide guidance for patients who are planned for the operation.

In the era of percutaneous interventional therapies for cardiac diseases, the high variability of coronary venous anatomy is a clinical focus and real challenge [11]. The presence, number, and course of the CS, GCV, PIV, and AIV are constant. By contrast, the presence, number, and course of the PVLV, LMV, and SCV are highly heterogeneous. The CS ostium is the primary point of entry into the coronary venous network. In our study, the size of the CS ostium in the anteroposterior direction was smaller than that in the superoinferior direction in all the patients, indicating that the ostium was oval. This finding was similar to those of Ma et al. [12] and Sun et al. [5] The diameters of the CS ostium and its tributaries of many patients suffering from chronic systolic heart failure or receiving coronary bypass grafts (CABG) have significantly enlarged [13–16]. This condition may be a consequence of the increased coronary venous blood flow and increased right atrial pressure in patients with heart failure. The increased coronary venous blood flow is related to the severity of heart failure [17, 18]. Mlynarski et al. [16] revealed that the distribution of pressures within the arterial and venous vessels of patients who receive CABG changed after the operation. A high pressure in the CVS can cause the expansion of veins as a compensation mechanism. Nevertheless, Van de Veire et al. [7] found that the diameters of the CS ostium and its tributaries do not change in patients with a history of MI compared with patients who have significant CAD without a history of MI and patients who have normal coronary arteries. In the present study, no significant differences in the diameters of the CS ostium and its tributaries were noted among the four groups. This result might suggest that the size of the CS ostium and its tributaries was not correlated with coronary artery calcification.

**Fig 3:**
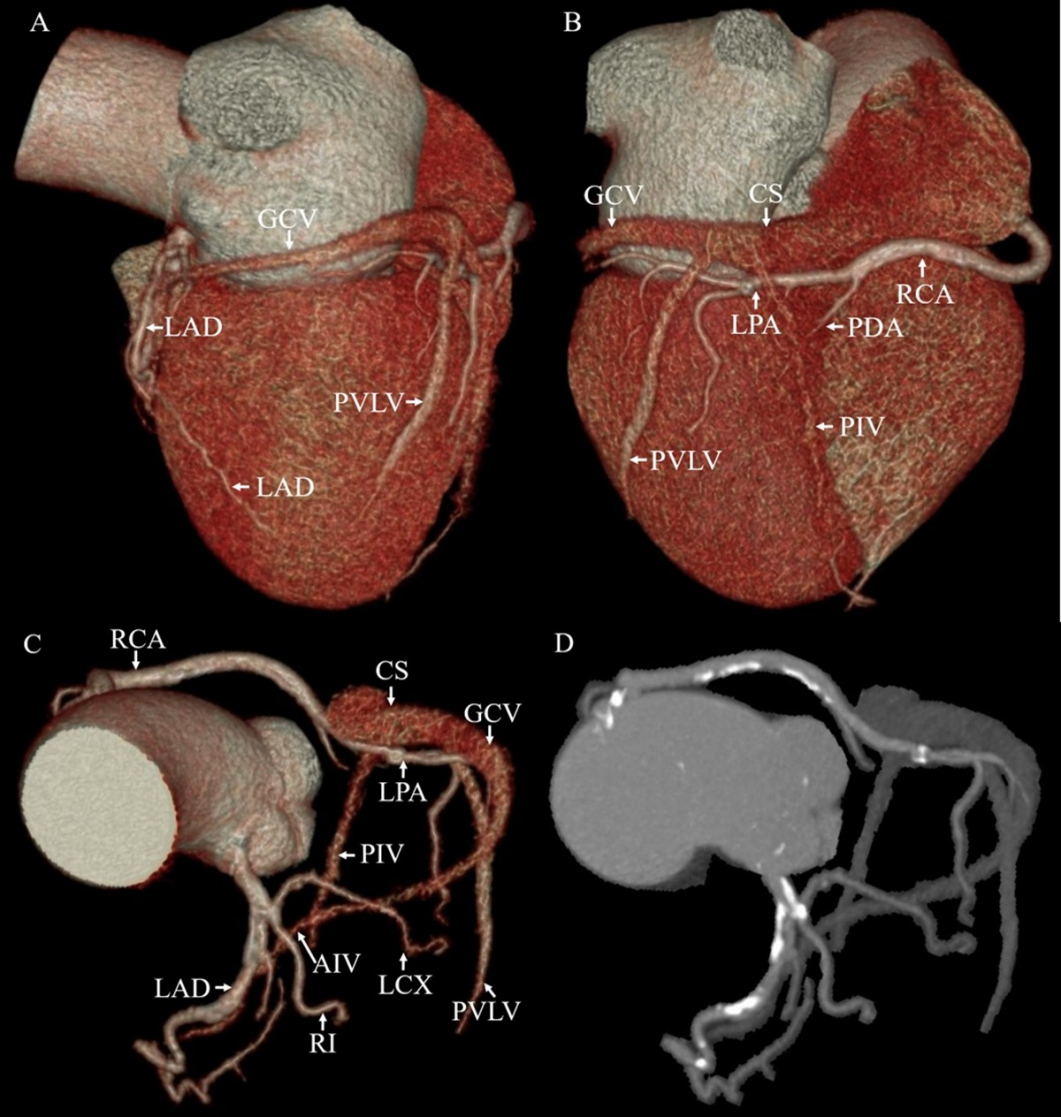
Volume-rendering images show absence of the left marginal vein (LMV) in a patient with CACS = 1201 AU. (A) Left lateral view of the heart; (B) posterior view of the heart; and (C) and (D) anterior views of the coronary tree. (D) shows the extensive calcification on the coronary artery. The coronary sinus (CS), great cardiac vein (GCV), posterior interventricular vein (PIV), posterior vein of the left ventricle (PVLV) and anterior interventricular vein (AIV) are visualized in this patient. Also note the left anterior descending artery (LAD), left circumflex coronary artery (LCX), ramus intermedius (RI), posterior descending artery (PDA), posterior branch of left ventricle artery (LPA) and right coronary artery (RCA).

The highest variability is observed in the number of tributaries between the PIV and AIV [5, 19–23]. Those tributaries mainly include PVLV and LMV. The prevalence of the PVLV and LMV varies in different studies when using various types of CT (Table 4). The PVLV and LMV are crucial for CRT because they are frequently used in the LV lead implantation. Evaluation of the coronary veins anatomy near the target region for potential LV lead implantation should be done to improve the success rate of CRT [24]. A recent study showed that the PVLV and LMV are less frequently present in CABG patients with reduced LV ejection fraction as compared to CABG patients with preserved LV ejection fraction, controls, and even non-ischemic cardiomyopathy [25]. Van de Veire et al. [7] who studied 100 subjects by using 64-slice CT and found the frequency of having no LMV was higher in patients with previous MI. These observations might indicate that the absence of LV tributaries in CAD patients with reduced LV ejection fraction or patients with a history of MI might preclude the transvenous placement of a LV lead and affect their CRT response. Our results were similar to those of Van de Veire et al [7]. We observed that the incidence of the LMV of the patients with coronary artery calcification was lower than that of the patients who had no calcification. ANOVA indicated no statistically significant differences in the prevalence of the LMV among the calcification groups. Fig 3 presents an example of an absent LMV in a patient with extensive coronary artery calcification. However, no statistically significant difference in the presence or absence of the PVLV was observed in the four groups in our study. Our findings might suggest that the coronary venous anatomy varied before MI happens. In this work, we also found that patients with coronary artery calcification had higher chance of having either one PVLV or LMV than patients without calcification. It also indicated that patients with coronary artery calcification had fewer visible coronary veins. This result was inconsistent with the findings of Mlynarska et al. [9], which may be due to the use of different detection equipment or the insufficient sample size. This is a controversial issue that needs further study. We observed that the total number of the PVLV and LMV ranged from 1 to 5 and only three patients had 5 branches. Randhawa et al. [26] examined 50 formalin fixed adult cadaveric hearts and found that the total number of the PVLV and LMV ranges from 1 to 4. to some extent, their findings were consistent with ours. Furthermore, the number of optimal tributaries draining the lateral LV wall should be determined because the fewer the number of tributaries is, the less the scope of the optimal site selection of LV lead implantation will be [27].

Some limitations were identified in our study. First, the 256-slice CT scans were tailored for the optimal visualization of coronary arteries. Thus, no special scan and contrast protocol for venous enhancement were used, and the coronary veins with small diameters could not be visualized. Second, the relationship between the calcification of each coronary artery and the anatomical variations of adjacent coronary veins was not specifically analyzed in the present study, which is what we will do in the future studies. Other limitation was that multislice CT involves the use of a particular radiation dose and a contrast medium. Their required amounts can be decreased by applying the radiation- and contrast-sparing protocol of CTA on 256-slice CT [28].

**Table 4:** Prevalence of the PVLV and LMV obtained from different studies that evaluated the coronary venous system using various kinds of CT.

	Mao et al. [19]	Tada et al. [20]	Abbara et al. [21]	Mlynarski et al. [22]	Genc et al. [23]	Sun et al. [5]
Imaging technique	EBCT	8-slice CT	16-slice CT	64-slice CT	128-slice CT	256-slice CT
Study population	231	70*	54	199	357§	102
PVLV, %	81	94	79. 6	62.3	87	77.5
LMV, %	78	84	92.6	80.4	87.9	66.7

*6 patients were not evaluated because of the poor image quality; §18 patients were not evaluated because of the poor image quality.

## Conclusion

The coronary venous anatomy varies in patients with coronary artery calcification. The LMV that constitutes one of the typical target veins for CRT may be less frequently present in patients with coronary artery calcification than in patients without coronary artery calcification, possibly making LV lead implantation more difficult during CRT. The pathophysiological mechanism of this finding should be further studied.
